# *De novo* pyrimidine synthesis is necessary for intestinal colonization of *Salmonella* Typhimurium in chicks

**DOI:** 10.1371/journal.pone.0183751

**Published:** 2017-10-17

**Authors:** Hee-Jeong Yang, Lydia Bogomolnaya, Michael McClelland, Helene Andrews-Polymenis

**Affiliations:** 1 Department of Microbial and Molecular Pathogenesis, College of Medicine, Texas A&M University System Health Science Center, Bryan, TX, United States of America; 2 Institute of Fundamental Medicine and Biology, Kazan Federal University, Kazan, Russia; 3 Department of Microbiology and Molecular Genetics, and Pathology and Laboratory Medicine, University of California, Irvine, CA, United States of America; New York State Department of Health, UNITED STATES

## Abstract

*pyrE* (STM3733) encodes orotate phosphoribosyltransferase (OPRTase; EC 2.4.2.10), the fifth enzyme of the *de novo* pyrimidine biosynthetic pathway. We identified a *ΔpyrE* mutant as under selection in screening of a *Salmonella* mutant library in 4-day old chicks. Here, we confirm that a *ΔpyrE* mutant colonizes 4-day old chicks poorly in competitive infection with isogenic wild type, and that the ability of this mutant to colonize chicks could be restored by providing a copy of *pyrE in trans*. We further show that our *ΔpyrE* mutant grows poorly in nutrient poor conditions *in vitro*, and that the ability of this mutant to grow is restored, both *in vitro* and in chicks, when precursors to the pyrimidine salvage pathway were provided. This finding suggests that the environment in the chick intestine during our infections lacks sufficient precursors of the pyrimidine salvage pathway to support *Salmonella* growth. Finally, we show that the colonization defect of a *ΔpyrE* mutant during infection occurs in to chicks, but not in CBA/J mice or ligated ileal loops in calves. Our data suggest that *de novo* pyrimidine synthesis is necessary for colonization of *Salmonella* Typhimurium in the chick, and that the salvage pathway is not used in this niche.

## Introduction

**Fig 1:**
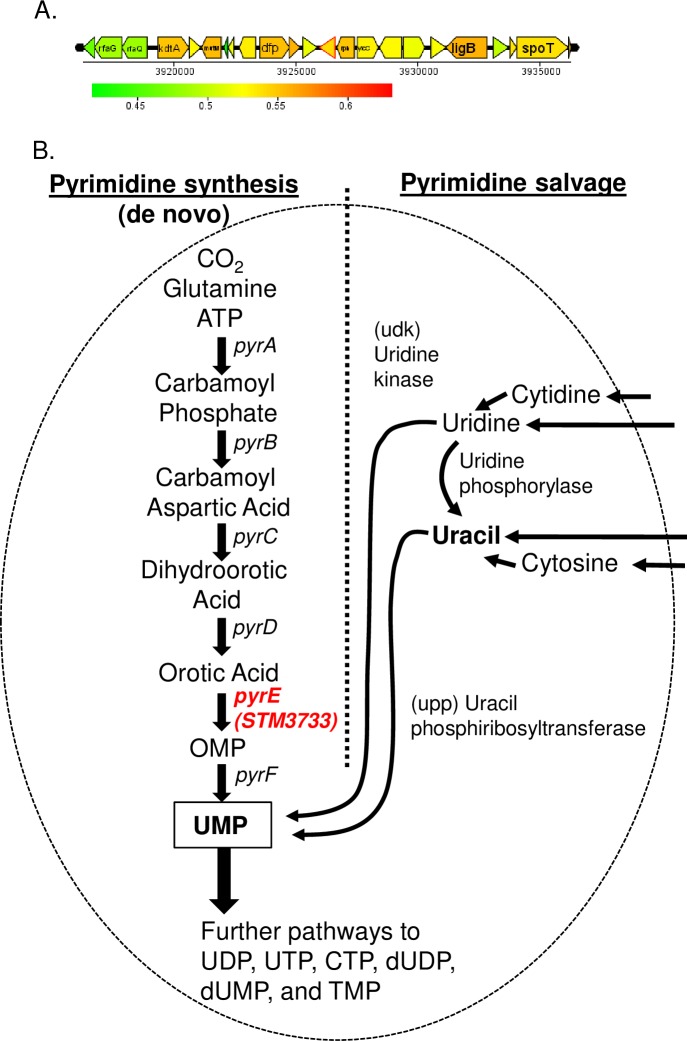
*pyrE* encodes fifth enzyme in pyrimidine biosynthesis. (A) Genetic context of *pyrE* (STM3733) in *Salmonella* Typhimurium. (B) *De novo* pyrimidine synthesis and salvage pathways in bacteria. Pathways that produce UMP, the precursor of all pyrimidines, are shown. The six steps of the *de novo* pyrimidine biosynthesis pathway and their corresponding enzymes, genes in parentheses are shown. Double arrowheads indicate that the activity is capable of both conversions.

Organisms can acquire pyrimidines by two mechanisms, they can either generate them *de novo*, or acquire them via a salvage pathway. *De novo* pyrimidine synthesis occurs in all organisms [1] and is accomplished by the sequential reactions of six enzymes (Fig 1): carbamyl phosphate synthetase (CPSase; EC 2.7.2.5), aspartate transcarbamylase (ATCase; EC 2.1.3.2), dihydroorotase (DHOase; EC 3.5.2.3), dihydroorotate dehydrogenase (DHOdehase; EC 1.3.3.1), orotate phosphoribosyltransferase (OPRTase; EC 2.4.2.10), and orotidylate decarboxylase (OMPdecase; EC 4.1.1.23) [2–4]. These six enzymes are encoded by six unlinked genes in all organisms [1, 5]. In the second mechanism, the pyrimidine salvage pathways converts preformed pyrimidine bases or nucleosides into pyrimidine nucleotides [6]. Enzymes including uridine kinase (udk, EC 2.7.1.48), UMP pyrophosphorylase (upp, EC 2.4.2.9), uridine phosphorylase (udp, EC 2.4.2.3), and cytidine deaminase (cdd, EC 3.5.4.5) are involved in this process [7].

In *Salmonella* Typhimurium, the common pyrimidine biosynthetic pathway consists [1] of six enzymes involved in the pathway that are encoded by six unlinked genes, *pyrA (STM0067)*, *pyrB (STM4460)*, *pyrC (STM1163)*, *pyrD (STM1058)*, *pyrE (STM3733) and pyrF (STM1707)* respectively (Fig 1) [5]. Several studies of the regulation of expression of the *pyr* genes and their role in virulence have been reported [8–12]. Fox and Bzik have reported that *de novo* pyrimidine synthesis is required for virulence of *Toxoplasma gondii* [11] and recently it has been reported that pyrimidine biosynthesis modulates production of biofilm determinants in *E*. *coli* [12].

We became interested in *pyrE (STM3733)* because we identified this gene as under selection in a genetic screen for mutants required for *Salmonella* Typhimurium infection of 4-day old chicks [13]. *pyrE* (*STM3733)*, encodes orotate phosphoribosyltransferase (OPRTase; EC 2.4.2.10), needed for the fifth step of pyrimidine biosynthesis and catalyzes the formation of orotidine 5’-monophosphate (OMP) from orotic acid (OA) and phosphoribosyl pyrophosphate (PRPP) [14]. In *Listeria monocytogenes pyrE* expression was induced preferentially when this organism was inside J774 murine macrophage-like cells, and the *Listeria pyrE* null is a pyrimidine auxotroph [15]. However, mutations in *pyrE* did not reduce virulence of *Listeria* in either C3H or BALB/C mice [15, 16]. In contrast, some transposon mutants of *Salmonella* Typhimurium that cannot survive within macrophages and are less virulent in *Salmonella*-sensitive BALB/C mice were also reportedly purine or pyrimidine auxotrophs [17]. There have been no studies to assess the *pyrE* requirements for colonization and persistence in other animal models.

We studied the role of *pyrE* in intestinal colonization of the chick by *Salmonella*. We confirm that a mutant lacking *pyrE* is severely impaired for colonization in 4-day old chicks from day 1 post infection, and determined that mutants in this gene are pyrimidine auxotrophs. We further show that the *pyrE* mutant colonizes other animal models similar to the wild type, and that uracil supplementation restored the ability of the *pyrE* mutant to colonize chicks.

## Materials and methods

### Bacterial strains and growth conditions

All strains in this study are derived from *Salmonella enterica* serovar Typhimurium ATCC14028 (Manassas, VA) and are listed in Table 1. Strains were routinely cultured in Luria-Bertani (LB) broth and agar plates supplemented with the appropriate antibiotics at 37^°^C. Bacterial cultures used to infect chicks were grown to stationary phase in LB broth at 41^°^C with aeration and were supplemented with the appropriate antibiotics. Antibiotics and other supplements were used at the following concentrations: 40 mg/L 5-bromo-4-chloro-3-indolyl phosphate (XP), 50 mg/L kanamycin (Kan), 100 mg/L nalidixic acid (Nal), and 100mg/L carbenicillin (Carb).

**Table 1:** Strains used in this study.

Strain	Genotype	Reference
HA383	*S*. *enterica* sv. Typhimurium ATCC14028s	ATCC
HA420	WT 14028s Nal^R^	[18]
HA431	HA420 *ΔphoN*::Kan^R^	[18]
HA877	HA420 *ΔphoN*::Nal^R^ pWSK29	This study
HA1293	*ΔSTM3733*::Kan^R^ pWSK29::*STM3733*	This study
HA1301	*ΔSTM3733*::Kan^R^ pWSK29	This study
HA1470	HA420 *ΔSTM3733*::Kan^R^	This study
pWSK29	Cloning vector, AmpR	[19]

### Testing growth *in vitro*

Growth was tested *in vitro* by quantifying individual growth of the mutant and the wild type HA431 (HA420 *ΔphoN*::Kan^R^), and competitive growth of these strains in co-culture. Both individual and co-culture assays were performed in LB and M9 media [20, 21]. For measuring individual growth in LB broth, overnight cultures of mutant and wild type were sub-cultured 1:100 into sterile LB supplemented with kanamycin. For testing individual growth in M9 broth, 1 ml of bacteria was collected from overnight cultures by centrifugation and bacterial pellets were washed twice in M9 broth. Washed bacteria were resuspended in 1 ml of M9 and a 1:50 dilution was made into fresh M9 supplemented with kanamycin. All sub-cultures were grown at 37^°^C with aeration for 24 hours. The number of bacterial colony forming units (CFU) was evaluated at 1, 2, 5, 7, and 24 hours of subculture by plating serial ten-fold dilutions in phosphate buffered saline (PBS) on LB agar supplemented with kanamycin.

For testing competitive growth, overnight culture of mutant and wild type (HA431) were mixed in 1:1 ratio. This equal mixture was sub-cultured in fresh LB or M9 broth supplemented with kanamycin. Overnight cultures of mutant and wild type were washed twice in M9 broth prior to being mixed in equal proportions and grown in M9 broth. At 1, 2, 5, 7 and 24 hours of sub-culture, aliquots were collected, serially diluted, and plated on LB agar containing appropriate antibiotics and supplements for enumeration of CFU. The ratio of recovered mutant to wild type was normalized by the input ratio to determine the competitive index. All experiments were done three times on three separate occasions. Mean bacterial number was calculated and converted logarithmically. Statistical significance was determined using Student’s two-tailed t-test with significance set at p < 0.05.

### Plasmid construction

A plasmid containing the intact *pyrE* gene *(STM3733)* and flanking regions was constructed in the following way. A fragment containing the *pyrE* gene and approximately 200 base pairs upstream as well as 50–100 base pairs downstream was amplified using following primers; STM3733EcoRIF, 5’- accgaattcgtacagggtaccgcggaagg-3’ and STM3733HindIIIR, 5’- accaagcttggtaaagcgccaccgggcaa-3’. PCR products were generated using *pfu Turbo* DNA polymerase (Agilent Technologies) in 50 μl volume, and an annealing temperature of 58^°^C for 30 cycles. PCR products were digested with EcoRI and HindIII as per manufacturer instructions (New England Biolabs, Ipswich, MA). Digested PCR products were ligated into pWSK29 previously cut with the same enzymes [19]. Each ligation was transformed into XL1-Blue and positive clones were selected on LB plates containing carbenicillin. Correct inserts were confirmed by restriction digest and sequencing.

### Uracil supplementation

A sterile uracil stock solution (1%, Acros Organics) was used to supplement M9 minimal media (20 mg/L) or chick diets (1% wt/wt). To supplement uracil to chick diets, 100 g of chick diet (Harlan Teklad, Madison, WI) was soaked in 100 ml of 1% uracil solution and permitted to air dry [22].

### Animal experiments

#### Ethics statement

This study was carried out in strict accordance with the recommendation in the Guide for the Care and Use of Laboratory Animals of the National Institutes of Health. The protocol was approved by Institutional Animal Care and Use Committee (IACUC) of Texas A&M University (AUP permits: 2010–038 (chicks), 2011–167 (mouse), 2012–095 (calf).

#### Chick hatching

Specific pathogen free (SPF) eggs were obtained from Charles River SPAFAS (North Franklin, CT). Eggs were incubated in an egg incubator (GQF Manufacturing Co.) at 38^°^C and with 58–65% of humidity for 21 days. Eggs were periodically rotated for the first 18 days and then moved to the hatching tray for the last 3 days pre-hatch [20]. Chicks were housed in a poultry brooder (Alternative Design Manufacturing, Siloam Springs, AR) at 32^°^C to 35^°^C with *ad libitum* access to tap water and irradiated lab chick diet (Harlan Teklad, Madison, WI).

#### Competitive infections in the chick

Four-day old specific pathogen free (SPF) White Leghorn chicks were used. *ΔpyrE* and wild type, HA431 (ATCC14028 *ΔphoN*::Kan^R^) [20] were grown to stationary phase in LB supplemented with kanamycin at 41^°^C with aeration, and mixed in 1:1 ratio. Groups of 5 chicks were orally infected with 1x10^9^ CFU of this mixed inoculum. Infected animals were monitored twice daily for signs of illness. On days 1, 3, and 9 post-infection, chicks were humanely euthanized. The ceca, cecal tonsil, spleen, liver, and bursa were excised, homogenized in phosphate buffered saline (1ml PBS for cecal tonsil, 3 ml for all other organs), serially diluted, and plated on LB agar plate supplemented with kanamycin and XP. Inactivation of *phoN*, encoding alkaline phosphatase, abolishes the ability to cleave 5-bromo-4-chloro-3-indolyl phosphate (XP) resulting in the formation of white colonies on LB plates supplemented with XP while *phoN+* strains appear blue. However mutations in *phoN* do not affect the ability of *S*. Typhimurium to colonize or persist of in murine or chick models [23] (and data not shown).

For the infection in chicks fed uracil supplemented diets, chicks were randomly divided into 3 groups after hatching as follows; (1) chicks fed uracil untreated diets (Ura -/-), (2) chicks fed uracil supplemented diets until day 3 post-infection and then switched to un-supplemented diets (Ura +/-), (3) chicks fed uracil supplemented diets throughout the study (Ura +/+). Chick body weights were measured before infection and at time of euthanasia determine weight gain.

#### Competitive infections in the *Salmonella*-resistant mice

Groups of five 8- to 10-week-old female CBA/J mice (Jackson Laboratory) were infected by gavage with mixture of a *ΔpyrE* mutant and the wild type, HA431 (ATCC14028 *ΔphoN*::Kan^R^) as described previously [18]. Briefly, strains were grown to stationary phase at 37^°^C with aeration and mixed in a 1:1 ratio of *ΔpyrE* to wild type. Five mice were infected with 1x10^9^ CFU of equal mixture of *ΔpyrE* and wild type and infected mice were observed daily for signs of illness. Feces were collected every 3 days, homogenized in sterile phosphate buffered saline (PBS), serially diluted and plated to enumerate colony forming units (CFU) of each strain. On day 41 post-infection, mice were euthanized. The cecum, Peyer’s patch, mesenteric lymph node, spleen and liver were excised, homogenized, serially diluted in PBS, and plated on LB supplemented with kanamycin and XP to enumerate CFU.

#### Competitive infection in ligated ileal loops in the calf

We purchased Angus cross calves from a breeding herd at the Veterinary Medical Park at Texas A&M University. Calves were separated from the dam at approximately 24 hours after birth, and measurement of total serum protein was used to evaluate adequate passive transfer. Calves were housed in an AAALAC-approved barn, fed milk replacer twice daily, and provided with water and grass hay. Selective fecal cultures were performed at least once weekly to ensure calves remained negative for *Salmonella* spp. [24] [25]. At 3 to 6 weeks of age, calves were anesthetized for ligated ileal loop surgery and surgery was performed as previously described [26] [27]. Ligated ileal loops were infected by intraluminal injection of 3 ml of a 1:1 mixture of the *ΔpyrE* mutant bearing the empty vector (HA1301, *ΔSTM3733*::Kan^R^ *pWSK29)* and wild type, HA877 (HA420 *ΔphoN*::Nal^R^ pWSK29) containing approximately 1x10^9^ CFU total. At 12 hours post infection, tissue, mucus, and luminal fluid were collected, homogenized, serially diluted in PBS, and plated to enumerate CFU. Calves were humanely euthanized by barbiturate overdose (pentobarbital) administered intravenously. These infections were performed in three different animals.

## Results

### *Salmonella* Typhimurium *ΔpyrE* mutant is attenuated during chick oral infection

We identified the *pyrE* gene as a candidate needed for colonization and persistence of *Salmonella* Typhimurium after oral infection of 4-day old chicks. *pyrE* encodes orotate phosphoribosyltransferase, the fifth enzyme involved in pyrimidine biosynthesis. PyrE catalyzes the formation of orotidine 5’-monophosphate (OMP) from orotate and phosphoribosyl pyrophosphate (Fig 1A) [14]. The genomic context of *pyrE* (*STM3733*) is shown in Fig 1B (annotated as orotate phosphoribosyltransferase (EC 2.2.4.10, OPRTase).

**Fig 2:**
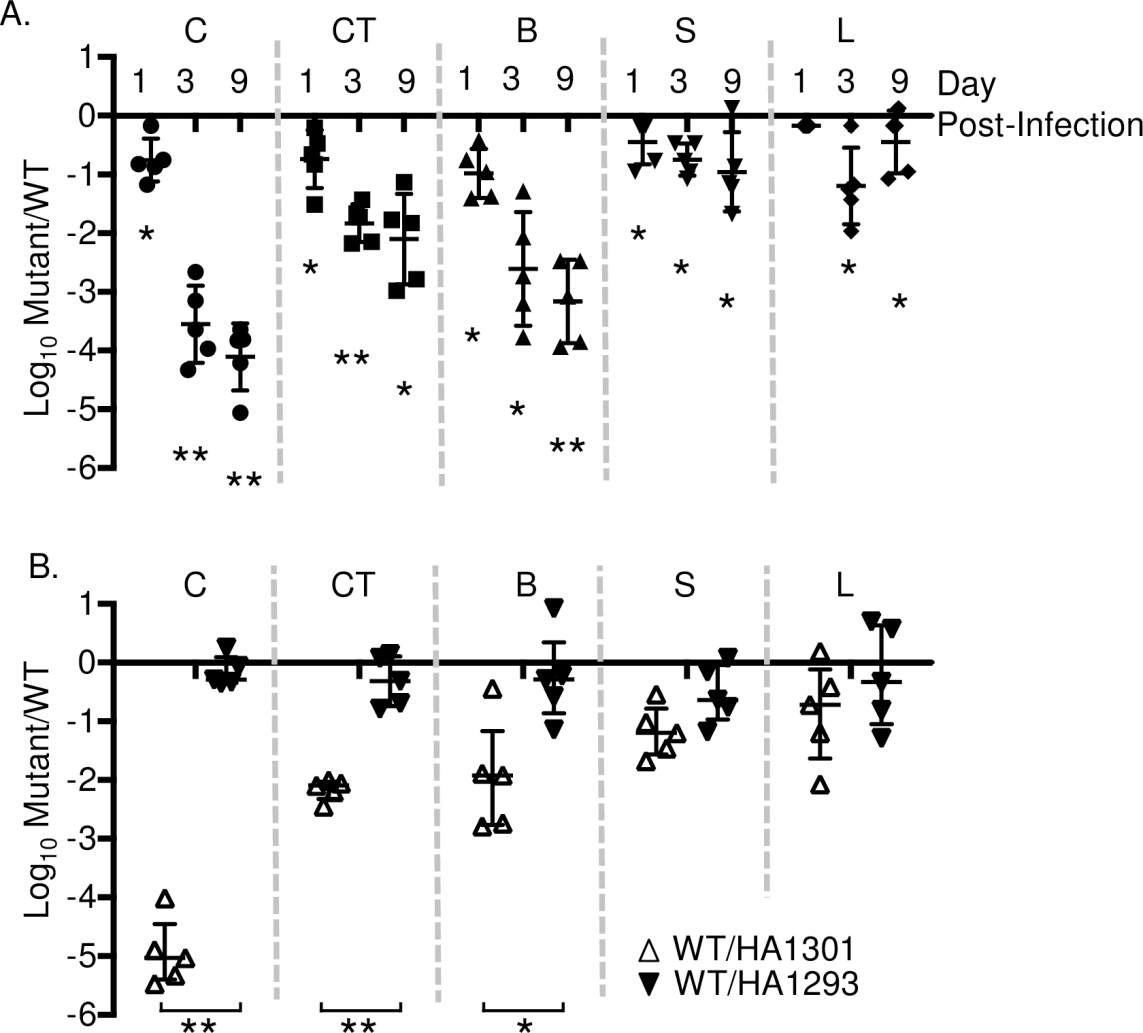
*ΔpyrE* mutant colonizes 4-day-old chicks poorly in competitive infection with wild type. (A) Fifteen 4-day-old chicks were infected orally with 1x10^9^ CFU of equal mixture of *ΔpyrE* and isogenic wild type, HA431 (HA420 *ΔphoN*::Kan^R^). At day 1, 3, and 9 post-infection, 5 chicks were humanely euthanized, ceca (C), cecal tonsil (CT), bursa (B), spleen (S), and liver (L) were excised, homogenized in PBS, and plated on LB agar plate supplemented with kanamycin and XP to enumerate the colony forming units (CFU) for *ΔpyrE* and wild type respectively. (B) Return of wild type copy of *pyrE in trans* restores colonization to the level of wild type during competitive infection with wild type, HA877 (HA420 *ΔphoN*::Nal^R^ pWSK29) and either HA1301(*ΔpyrE*::Kan^R^ pWSK29) (open trianges) or HA1293 (*ΔpyrE*::Kan^R^ pWSK29::STM3733) (filled trianges) at day 9 post-infection. Infections were performed as described in (A). Data are shown as the ratio of mutant to wild type recovered from each tissue normalized to the input ratio and converted logarithmically. Horizontal bars denote the mean, and error bars denote the standard deviation. Statistical significance was determined by using a Student’s 2-tail *t*-test with p < 0.05 (*), p< 0.001 (**).

To confirm the requirement for *pyrE* during colonization and persistence in this animal model, we retested the Δ*pyrE* mutant in chicks in competitive infection with isogenic wild type, HA431 (ATCC14028 *ΔphoN*::Kan^R^). A mutant in *pyrE* colonized all organs tested poorly from the earliest points in infection. The *pyrE* null mutant had a significant reduction in colonization of all organs that was most severe in the gastrointestinal tract with greater than a 1000-fold reduction relative to the wild type at day 3 post infection (Fig 2A). Returning an intact copy of *pyrE in trans* to a *ΔpyrE* mutant reversed the colonization defect of the deletion mutant in chicks in all organs we examined (Fig 2B). Thus, we confirmed that enzymes needed for *de novo* pyrimidine synthesis in *Salmonella* Typhimurium are needed for colonization of 4-day old chicks. In addition, we infer that precursors of the salvage pathway are either not present or not available in high enough quantity to support the growth of the *pyrE* mutant in chicks.

### *ΔpyrE* mutant in Typhimurium is defective for growth in M9 minimal media

**Fig 3:**
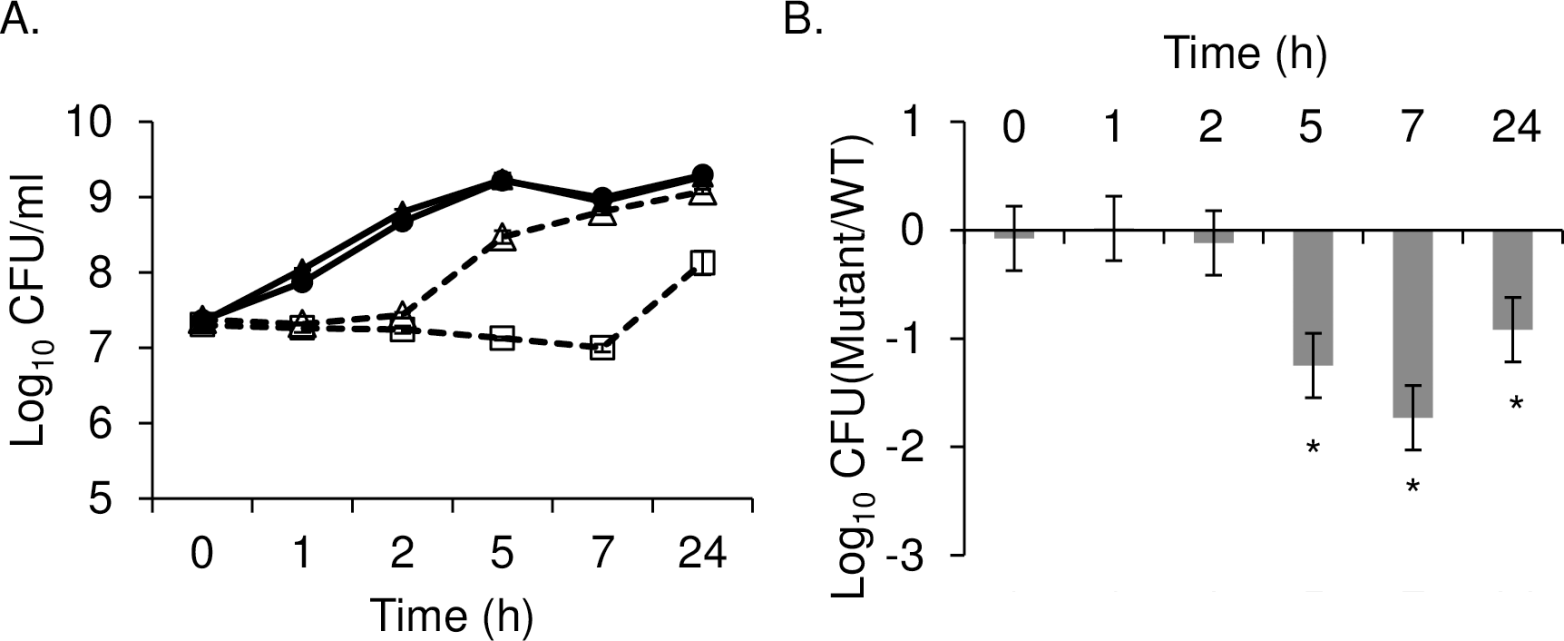
*ΔpyrE* mutant grows poorly in M9 minimal media. Growth was tested *in vitro* by individual growth (A) or competitive growth with wild type (B). (A) Stationary phase cultures of the *ΔpyrE* mutant (open square) and wild type, HA431 (HA420 *ΔphoN*::Kan^R^) (dark grey triangle) were sub-cultured in LB (solid line) or M9 broth (dotted line) supplemented with kanamycin for 24 hours. Overnight cultures were washed in M9 broth twice prior to sub-culturing in M9 broth. At the indicated time points, CFU in each culture was enumerated after serial dilution and plating. (B) Overnight cultures of *ΔpyrE* mutant and the wild type (HA431) were washed twice with M9 broth and mixed at 1:1 ratio in M9 broth. Resulting mixture was sub-cultured in M9 broth supplemented with kanamycin at 37^°^C for 24h. At the indicated time points the CFU of both strains was enumerated by serial dilution and plating. Dataare shown as ratio of mutant to wild type recovered normalized by the input ratio, and converted logarithmically. Statistical significance was determined by using a Student’s two-tail *t*-test with p*<* 0.05 (*) and error bars denote standard error. Experiments were performed on three independent occasions.

Previous studies using *L*. *monocytogenes* showed that *pyrE* mutants in *Listeria* are pyrimidine auxotrophs [15, 28]. Thus, the inability of a *ΔpyrE* mutant of *S*. Typhimurium to colonize chicks could be due to a growth defect under pyrimidine-limited conditions. In order to test this hypothesis, we measured growth of Typhimurium *ΔpyrE* mutant in LB and M9 minimal broth. The *ΔpyrE* mutant grew indistinguishably from wild type in LB, but grew poorly in M9 broth (Fig 3A). The growth defect of the *ΔpyrE* mutant was also observed in competitive growth with isogenic wild type, HA431 in M9 broth. The *ΔpyrE* mutant maintained the same cell density as the wild type during first 2 hours of subculture but a growth defect became apparent at 5 hours (Fig 3B). The *ΔpyrE* mutant grew approximately 10-fold less than the wild type at 24 hours and this was consistent with our other results.

### Uracil supplementation reverses growth defect of *ΔpyrE* mutant in M9 media

**Fig 4:**
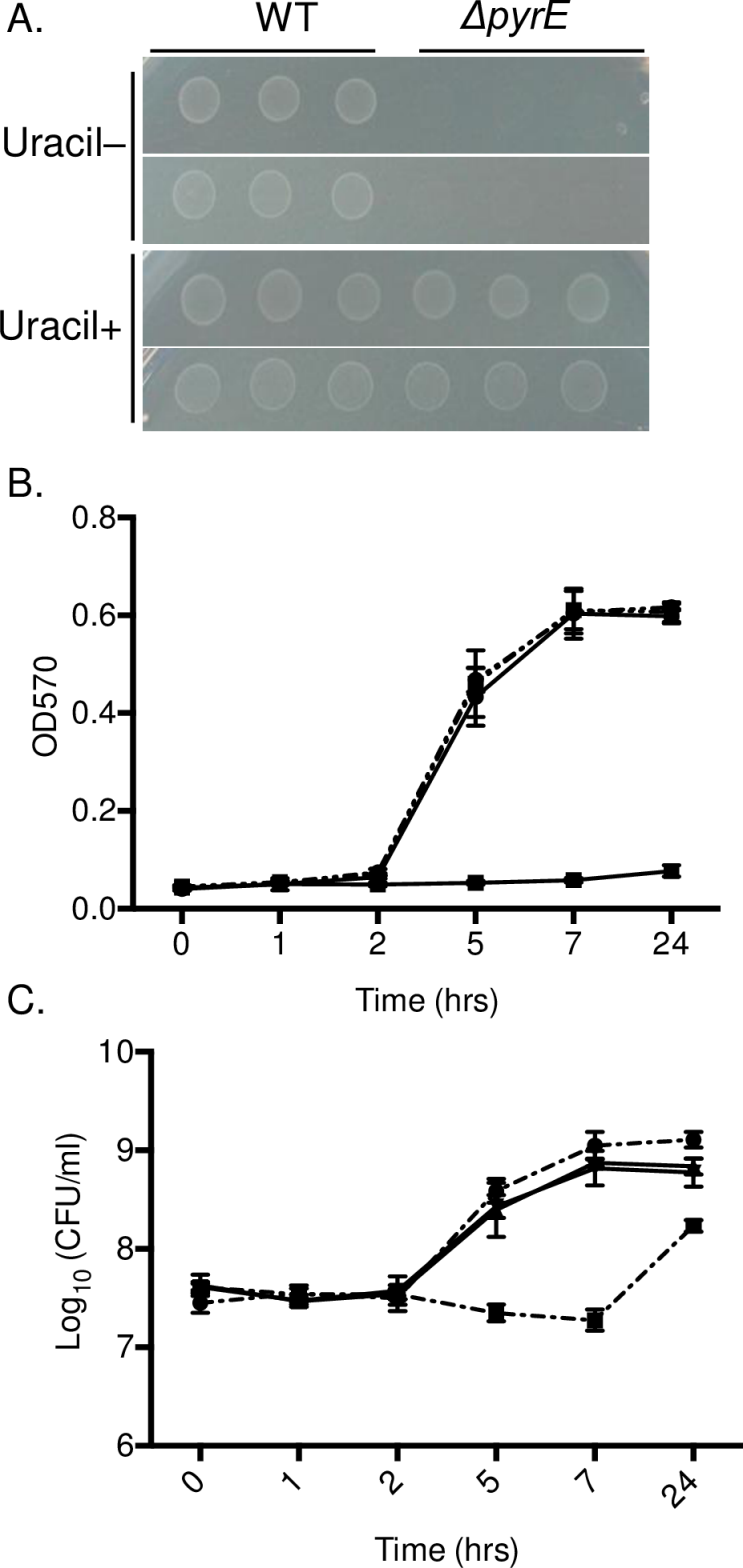
Growth defect of *ΔpyrE* mutant in M9 media is restored by uracil supplementation. (A) Overnight cultures of a *ΔpyrE* mutan*t* and the wild type, HA431 (HA420 *ΔphoN*::Kan^R^) were washed in M9 minimal broth twice and 3 μl of washed culture was spotted on M9 media with (Uracil+) or without uracil supplementation (Uracil-) (20 mg/L). Plates were incubated overnight at 37^°^C. This experiment was performed in triplicate and representative photographs are shown. (B-C) Bacteria (*ΔpyrE* mutant: filled square, or the wild type wild type, HA431 (HA420 *ΔphoN*::Kan^R^): filled circle) from overnight cultures were washed in M9 broth, and sub-cultured in un-supplemented M9 broth with (solid line) or in M9 broth with uracil supplementation at 20mg/ml (dashed line). At the indicated time points, aliquots were taken for absorbance measurement at 570nm (B), or were serially diluted in PBS and plated on LB supplemented with kanamycin and XP to enumerate CFU (C). Bars indicate mean and error bars indicate standard deviation. All experiments were performed on three separate occasions.

To investigate whether the growth defect of *ΔpyrE* mutant in M9 minimal media is due to the lack of active *de novo* pyrimidine synthesis pathway, we examined whether the salvage pathway could rescue the growth defect of the *ΔpyrE* mutant in M9 media. We supplemented solid or liquid M9 minimal media with 20 mg/L of uracil, which can be converted to UMP through the pyrimidine salvage pathway (Fig 1A). Addition of uracil to the M9 plates restored the ability of the *ΔpyrE* mutant to grow to levels similar to the wild type organism (Fig 4A). We observed similar results when the wild type and the *ΔpyrE* mutant bacteria were grown in M9 broth supplemented with uracil (Fig 4B and 4C). Growth of the *ΔpyrE* mutant was nearly equal to wild type in uracil supplemented M9 broth (Fig 4B and 4C). These results support the hypothesis that the growth defect of the *ΔpyrE* mutant in pyrimidine limited conditions is due to restriction of *de novo* pyrimidine synthesis, and this growth defect can be rescued when the salvage pathway is activated through uracil supplementation. This result is consistent with the previous study by Klarsfeld et al. [15].

### Colonization defect of *Salmonella* Typhimurium *ΔpyrE* mutant is restricted to chickens

**Fig 5:**
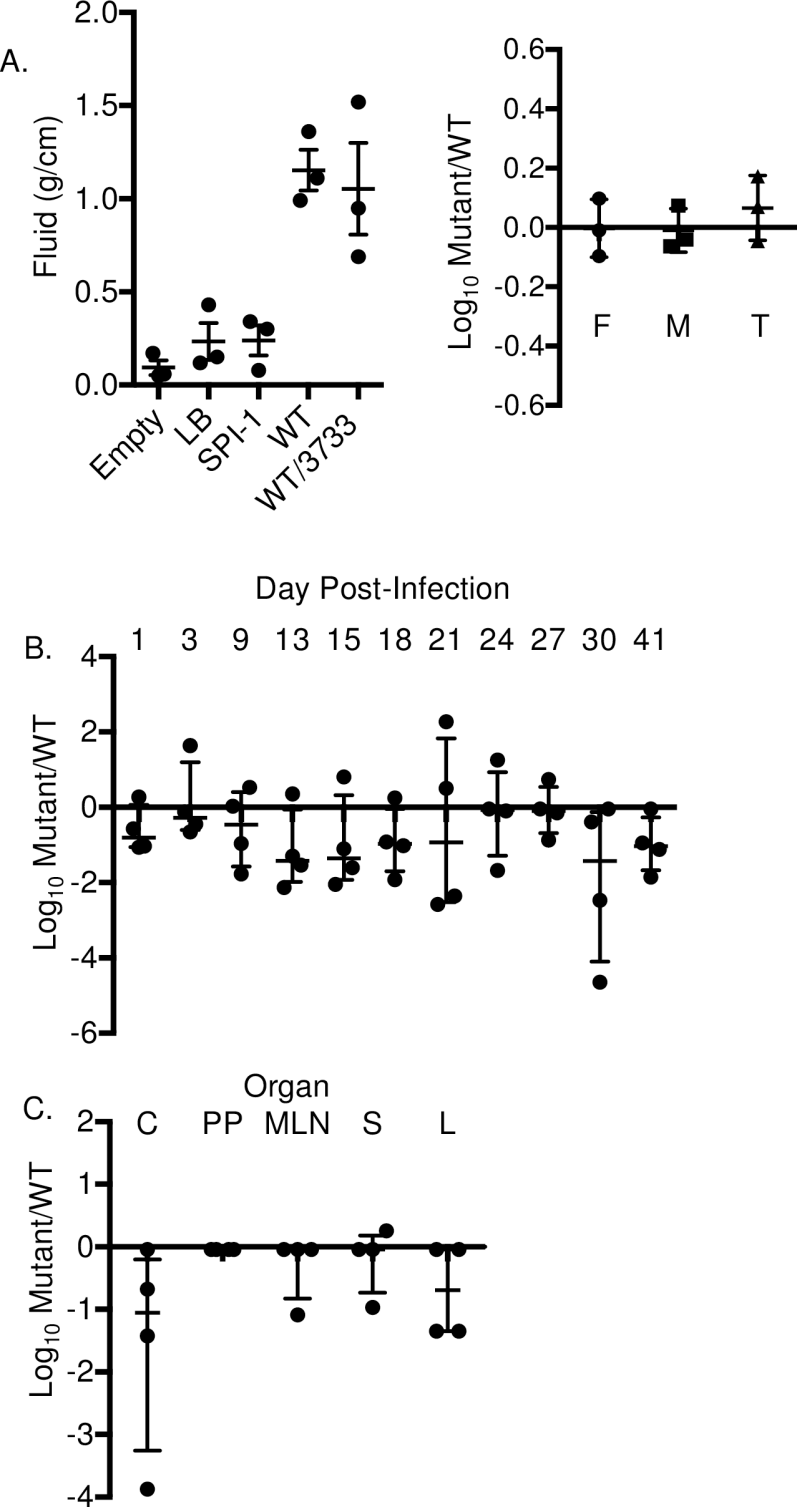
*ΔpyrE* mutant is able to colonize and persist in mice and calves in the levels similar to wild type. (A) Bovine ligated ileal loops were infected with 1x10^9^ CFU of a 1:1 mixture of the *ΔpyrE* mutant and wild type, HA877 (HA420 *ΔphoN*::Nal^R^ pWSK29) by intraluminal injection. After 12 hours post-infection loops were excised and the amount of luminal fluid was determined as a gross measure of inflammation. Luminal fluid (F), mucus (M) and tissue (T) were collected and processed for CFU determination. Infection was performed in 3 different animals. (B) Four CBA/J mice were infected with 1x10^9^ CFU of a 1:1 mixture of the *ΔpyrE* mutant and wild type, HA431 (HA420 *ΔphoN*::Kan^R^) by gavage.) Feces were collected every three days, and the CFU of each strain was determined by serial dilution and plating. (C) At 41 days post-infection, the cecum (CC), Peyer’s patches (PP), mesenteric lymph nodes (MLN), spleen (S), and liver (L) were excised, and processed as described in Fig 1. Data is shown as the ratio of mutant to wild type recovered, normalized to the input ratio, and converted to logarithmically. The median is indicated. Statistical significance was determined by using a Student’s two-tail *t*-test with p < 0.05.

Previously it has been reported that a *ΔpyrE* mutant in *Listeria monocytogenes* does not have reduced virulence in mice [15, 16]. We show that a *ΔpyrE* mutant in *S*. Typhimurium colonizes 4-day old chicks very poorly (Fig 2A). We wanted to further investigate the ability of *S*. Typhimurium *ΔpyrE* mutants to survive in other animal models. First, we tested the ability of the *ΔpyrE* mutant to survive intestinal inflammation during a 12 hour incubation in ligated ileal loops in calves, a model that closely resembles the early stages of human infections with non-typhoidal *Salmonella*. We found that survival of the *ΔpyrE* mutant was very similar to the wild type organism in ligated ileal loops, in the face of robust fluid generation in the loop (Fig 5A, Left panel = fluid generation in the loop, Right panel = mutant survival relative to WT survival). This data suggested that disruption of pyrimidine biosynthesis does not limit *Salmonella* survival and growth in intestinal fluid, mucus or tissue during short-term infections.

We also infected *Salmonella*-resistant CBA/J mice with an equal mixture of the *ΔpyrE* mutant and isogenic wild type, HA431 (ATCC14028 *ΔphoN*::Kan^R^), and monitored shedding of these strains in feces to 41 days post-infection (Fig 5B and 5C). In contrast to infection in chicks, the *ΔpyrE* mutant was able to colonize the intestinal tract of CBA/J mice well, and was shed in feces at levels similar to the wild type between 1 and 9 days post-infection. At 13 days post-infection and beyond, the ability of this mutant to colonize the intestinal tract in mice, although it appears to be reduced relative to the wild type, is not statistically significantly different from the wild type organism (Fig 5B). At 41 days post infection, the ability of the *ΔpyrE* mutant to colonize the ceca, Peyer’s patches, mesenteric lymph nodes, spleen and liver in infected CBA/J mice was not statistically significantly different from the wild type organism. Our data suggest that pyrmidine biosynthesis by the *de novo* pathway is not required during non-typhoidal *Salmonella* infection in mice, in contrast to the requirement for this pathway during infection in chicks.

### Uracil-supplemented diet rescues colonization defect of *Salmonella* Typhimurium *ΔpyrE* mutant in 4-day old chicks

A previous study has reported that the enzyme activity of carbamyl phosphate synthetase (CPSase; EC 2.7.2.5) and aspartate transcarbamylase (ATCase; EC 2.1.3.2), the first 2 enzymes in *de novo* pyrimidine synthesis are extremely low in 3-week-old chicks compared to other animals such as mice, rats or pigeons [29]. We hypothesized that the host specific colonization defect of *ΔpyrE* mutant in chickens is linked to insufficient pyrimidine precursors or synthesis in the chick. We randomly divided chicks into two groups immediately after hatching and each group was fed either uracil supplemented (uracil +) or regular (uracil -) diets, respectively. At day 4 post-hatch, both groups of chicks were infected with equal mixtures of *ΔpyrE* mutant and isogenic wild type, HA431.

**Fig 6:**
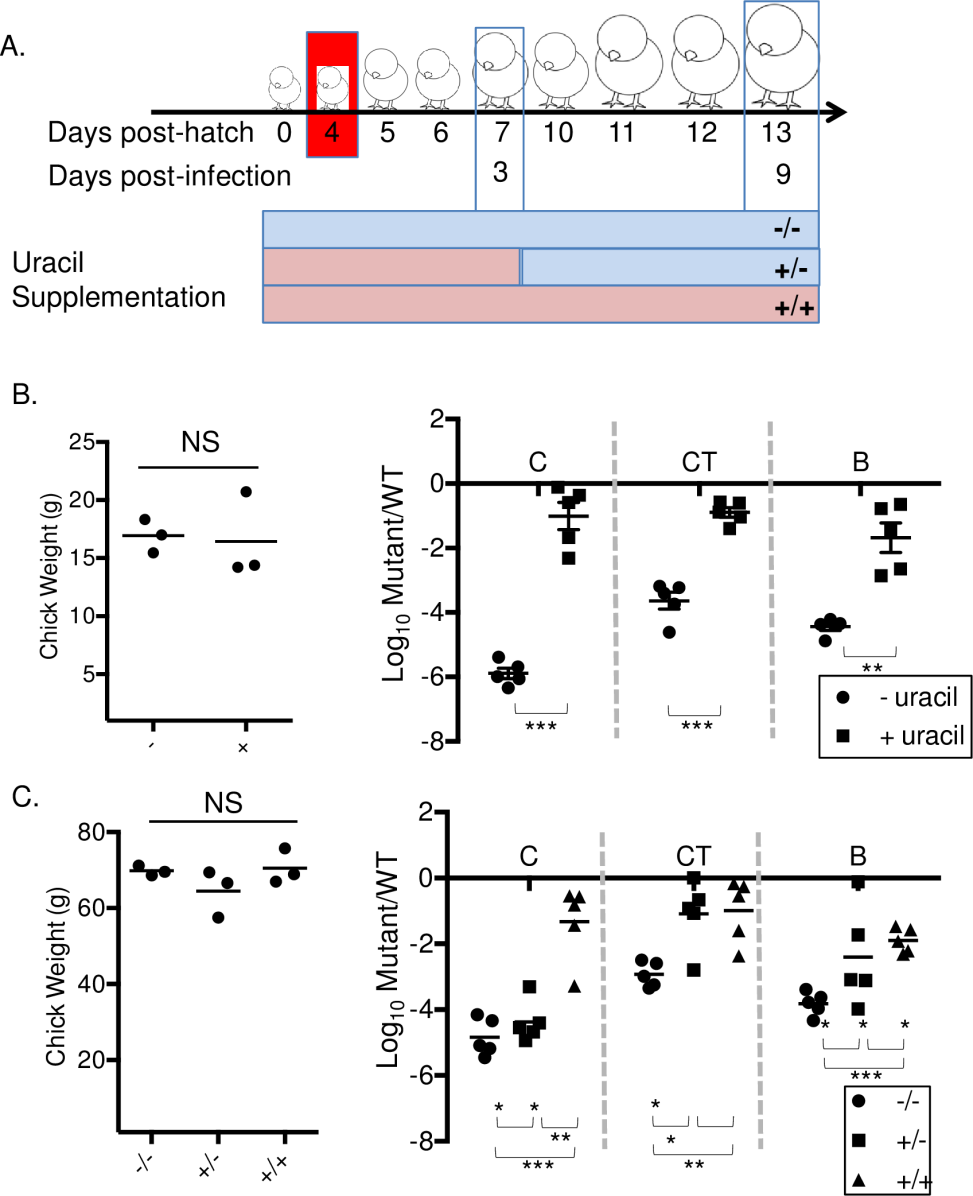
Colonization defect of *ΔpyrE* mutant in chicks is rescued by feeding uracil-supplemented diet. **(A)** Schematic of the experimental design. Chicks were divided into 2 groups after hatching and fed either our regular chick diet (no added uracil, or uracil -) or the same diet supplemented with uracil (uracil +). At day 4 post-hatch, chicks in both groups were weighed and orally infected with 1 x 10^9^ CFU of a 1:1 mixture of the *ΔpyrE* mutant and wild type, HA431 (HA420 *ΔphoN*::Kan^R^). **(B)** At day 3 post-infection (corresponds to 7 days of age), chicks from both groups were weighed and five chicks from each group were euthanized. **(C)** The remaining chicks being fed our regular chick diet were maintained on this diet for the duration of the experiment (uracil -/-, filled circles). The remaining chicks in uracil supplemented group were sub-divided into 2 groups: One group (uracil +/+, filled triangles) was fed a uracil-supplemented diet for the duration of our study, and the other group was returned to our regular chick diet (uracil +/-, no added uracil from days 3–9 post infection, filled squares). At day 9 post-infection, all three groups of chicks were weighed and euthanized. For all groups and all time points, ceca (C) cecal tonsil (CT) and Bursa of Fabricius (B) were collected and processed as described previously. Data are shown as ratio of mutant to wild type in the infected tissue, normalized to the input ratio and converted logarithmically. Statistical significance was determined by using a Student’s 2-tailed *t*-test with p < 0.05 (*), p< 0.01 (**), p< 0.001 (***), NS: not significant. Error bars denote standard error.

At day 3 post-infection, chicks from both groups were euthanized and various organs were collected and processed to assess *Salmonella* colonization. At this point, remaining chicks from the uracil-supplemented group were subdivided into 2 groups. The first group was maintained on uracil-supplemented food (uracil +/+) while the second group had uracil withdrawn from their diets (uracil +/-). At day 9 post-infection, these remaining chicks were euthanized and organs were collected and processed to compare *Salmonella* colonization between groups (Fig 6A outlines this scheme).

Uracil supplementation is well tolerated by chicks of this age, as uracil supplementation did not affect weight gain or general health of the chicks (data not shown). As expected, the *ΔpyrE* mutant colonized chicks fed regular diets poorly. However, the colonization defect of the *ΔpyrE* mutant was reversed in chicks fed diets supplemented with uracil in ceca, cecal tonsil, and bursa at day 3 post-infection (Fig 6B). Moreover, the *ΔpyrE* mutant was able successfully compete with wild type for colonization of all organs on Day 9 post infection in chicks continuously fed uracil supplemented diets (Fig 6C triangles). Finally, withdrawal of uracil from the chick diet at day 3 post-infection (Uracil +/-) resulted in an immediate decrease in colonization by the *ΔpyrE* mutant in ceca but not in cecal tonsil and bursa (Fig 6D square marks). These data support the hypothesis that pyrimidine supply is limited in the chick gastrointestinal tract, and that precursors of the salvage pathway are also not readily available.

## Discussion

The synthesis of pyrimidine nucleotides occurs through two distinct pathways: via pathway for *de novo* synthesis of pyrimidines from small molecules, or by a salvage pathway that utilizes preformed pyrimidine bases or nucleosides. The *de novo* pyrimidine biosynthetic pathway is highly conserved between animals and microorganisms [6] and it is accomplished by the sequential reaction of six enzymes that are encoded by six unlinked genes in *Salmonella* Typhimurium [1]. Both a previous genetic screen we performed, and a screen performed by others suggested that genes in the de novo pyrimidine synthesis pathway were under selection during colonization of chicks [30]. We now show that *de novo* pyrimidine synthesis in *Salmonella* is critical for this organism to colonize the intestine in chicks.

Our mutant lacking *pyrE*, which behaves as a pyrimidine auxotroph *in vitro*, has a severe colonization defect in this chicks. The growth defect of our *ΔpyrE* mutant in minimal media is reversed when uracil is provided and the salvage pathway can be used instead (Figs 3 and 4). Consistent with our findings, *Salmonella* mutants lacking *pyrD (STM1058)*, the fourth enzyme in the pyrimidine biosynthetic pathway encoding dihydroorotate dehydrogenase (DHOdehase; EC 1.3.3.1), also grow poorly in pyrimidine limited conditions [31], and appear to be under selection during a genetic screen 1-day old chicks [32]. Furthermore, *pyrC*, the third enzyme in the pyrimidine biosynthetic pathway (DHOase; EC 3.5.2.3) (Fig 1B), is up-regulated in cecal tissue harvested from chickens infected with *Salmonella* Typhimurium [33]. Our findings and those of others suggest that in young chicks, the *de novo* pathway for pyrimidine biosynthesis is critical for the establishment of non-typhoidal *Salmonella* infection.

Although our *pyrE* deletion mutant colonized chicks very poorly, it appears to colonize ligated ileal loops in calves and orally infected CBA/J mice very similar to the wild type organism. The activities of *pyrA* and *pyrB*, encoding carbamyl phosphate synthetase (CPSase; EC 2.7.2.5) and aspartate transcarbamylase (ATCase; EC 2.1.3.2), respectively, differ in various animals including rat, mice, pigeon and chick but are especially low in 3-week old chicks compared to other animal models, supporting our findings [29]. Additional work on *pyrE* mutants in *Listeria monocytogenes* also indicated that *pyrE* is not involved in virulence and colonization of *Listeria* in C3H and BALB/c mice [15]. These findings support the hypothesis that the chick intestine, but not the murine intestine, is a pyrimidine-limited. Furthermore, the salvage pathway for pyrimidine synthesis does not appear to be functional in young chicks, perhaps because precursors such as uracil may not be present in the intestine in high enough quantity.

Finally, the colonization defect of our *ΔpyrE* mutant was rescued by the addition of uracil to the chick diets over the 9-day infections we performed. While the *ΔpyrE* mutant colonized similar to the wild type when the diet was supplemented with uracil, uracil deprivation after the establishment of infection resulted in the immediate loss of the ability of the *ΔpyrE* mutant to colonize the intestinal tract. However, withdrawal of uracil supplementation after the establishment of infection in the presence of uracil did not affect the ability of the *pyrE* mutant to colonize and persist in the cecal tonsil and bursa. These results are in agreement with previous findings that a *ΔpyrE* mutant in *Listeria monocytogenes* is pyrimidine auxotroph, and uracil supplementation restores the growth of this mutant to wild type levels [15].

To summarize, we show that a *ΔpyrE* mutant in *Salmonella* Typhimurium does not grow in pyrimidine-limited media, and poorly colonized in 4-day old chicks. Reduced growth of the *ΔpyrE* mutant in pyrimidine-limited conditions could be restored through addition of uracil, which can be utilized in a salvage pathway to generate pyrimidines. Moreover, it appears that pyrimidine biosynthesis is not limiting to *Salmonella* growth during infection of every animal model, suggesting that one could use auxotrophies to probe the nutritional environment of different niches during infection.

## Supporting information

S1 File Raw data supporting the experiments described herein is contained in the S1 File. (XLSX)
